# Timed topical dexamethasone eye drops improve mitochondrial function to prevent severe retinopathy of prematurity

**DOI:** 10.21203/rs.3.rs-4619093/v1

**Published:** 2024-06-25

**Authors:** Hitomi Yagi, Myriam Boeck, Mariya Petrishka-Lozenska, Pia Lundgren, Taku Kasai, Gael Cagnone, Chaomei Wang, Jeff Lee, Yohei Tomita, Sasha A. Singh, Jean-Sébastien Joyal, Masanori Aikawa, Kazuno Negishi, Zhongjie Fu, Ann Hellström, Lois E.H. Smith

**Affiliations:** Boston Children’s Hospital; Boston Children’s Hospital; University of Gothenburg; University of Gothenburg; Brigham Women’s Hospital; CHU Sainte-Justine, Université de Montréal; Boston Children’s Hospital; Boston Children’s Hospital; Boston Children’s Hospital; Keio University School of Medicine; Brigham Women’s Hospital; CHU Sainte-Justine, Université de Montréal; Brigham Women’s Hospital; Keio University School of Medicine; Boston Children’s Hospital; University of Gothenburg; Boston Children’s Hospital

**Keywords:** Retinopathy of prematurity, oxygen-induced retinopathy, neovascularization, dexamethasone, eye drop, mitochondrial function

## Abstract

Pathological neovascularization in retinopathy of prematurity (ROP) can cause visual impairment in preterm infants. Current ROP treatments which are not preventative and only address late neovascular ROP, are costly and can lead to severe complications. We showed that topical 0.1% dexamethasone eye drops administered prior to peak neovessel formation prevented neovascularization in five extremely preterm infants at high risk for ROP and suppressed neovascularization by 30% in mouse oxygen-induced retinopathy (OIR) modeling ROP. In contrast, in OIR, topical dexamethasone treatment before any neovessel formation had limited efficacy in preventing later neovascularization, while treatment after peak neovessel formation had a non-statistically significant trend to exacerbating disease. Optimally timed topical dexamethasone suppression of neovascularization in OIR was associated with increased retinal mitochondrial gene expression and decreased inflammatory marker expression, predominantly found in immune cells. Blocking mitochondrial ATP synthetase reversed the inhibitory effect of dexamethasone on neovascularization in OIR. This study provides new insights into topical steroid effects in retinal neovascularization and into mitochondrial function in phase II ROP, and suggests a simple clinical approach to prevent severe ROP.

## Introduction

Retinopathy of prematurity (ROP) is a retinal neurovascular disorder in preterm infants who are born before completing retinal neural and vascular development, into a relatively hyperoxic extrauterine environment, particularly with supplemental oxygen treatment. Hyperoxia suppresses physiologic retinal vascular development, and recently formed capillaries may be lost (vaso-obliteration, Phase I ROP). As the neural retina slowly develops after birth, the avascular retina without an oxygen and nutrient supply becomes ischemic, driving pathological neovascularization in part through excess vascular endothelial growth factor (VEGF) (Phase II ROP)[1, 2]. Current treatments for Phase II ROP have limitations. Laser-photocoagulation destroys avascular retina. Intravitreal injection of anti-VEGF is costly, has a high rate of neovascularization recurrence and the drug may persist for weeks to months in the systemic circulation[3], potentially inhibiting vascularization of other developing organs[4]. There is an urgent need to develop effective, inexpensive and safe preventative treatments for ROP.

Dexamethasone is a glucocorticoid agonist. There are many mixed reports of glucocorticoids suppressing (and some enhancing) retinal neovascularization, but the detailed mechanism of dexamethasone suppression of pathological neovascularization is unknown. Importantly, the effects of dexamethasone on preventing neovascular ROP may depend on the route of administration, timing, and dose[5], which require a better understanding of the mechanisms that control the development of ROP Clinical and experimental evidence suggests that dexamethasone exerts anti-angiogenic functions[6–9]. Dexamethasone also regulates metabolism, and has the potential to protect against glial and neuronal apoptosis[10–14] and to suppress microglial reactivity[15] and resulting inflammation.

Topical dexamethasone eye drops are readily available worldwide and unlike laser and intravitreal anti-VEGF are easy to administer. If shown to be effective in preventing neovascular ROP, this treatment might fulfill a great need in both underdeveloped countries where the incidence of ROP is increasing rapidly as well as in countries with more advanced neonatal care. From a clinical perspective, topical dexamethasone administration may avoid adverse effects of systemic or intravitreal corticosteroid injection.

We conducted a small prospective pilot clinical study, using topical 0.1% dexamethasone eye drops in Type 1 ROP[16] in extremely preterm infants (gestational age 22 to 27 weeks) at very high risk for ROP This treatment, administered prior to the development of severe ROP requiring treatment, prevented ROP progression, promoted normal retinal vascularization, and prevented the need for laser or anti-VEGF treatment in all 5 cases. This was also reported in a small retrospective study.[17] To understand the mechanisms and timing, we used the well-known mouse model of ROP, oxygen-induced retinopathy (OIR)[18], and demonstrated that topical 0.1% dexamethasone eye drops administered daily prior to peak neovessel formation optimally suppressed neovascularization at P17. Treatment prior to any neovessel formation showed minimal prevention of neovascularization. Treatment after peak neovessel formation was ineffective at reducing neovascularization with a trend to increasing neovascularization. We found that topical dexamethasone eye drops suppressed neovascularization by modulating retinal mitochondrial function. This study provides new therapeutic strategies for ROP and new insights into mitochondrial control of Phase II ROP.

## Methods

### Patients.

A pilot prospective clinical study was performed to evaluate topical 0.1% dexamethasone eye drops in patients with ROP. Dexamethasone was prescribed off label, and this study was approved by the local ethics committee (Dnr 2019–02321, Registration date 2019-05-02) and performed following the committee regulations. It adhered to the Declaration of Helsinki for human research.

The inclusion criteria were the first sign of Type 2 ROP with stage 3, zone II without plus disease, i.e., the beginning of neovascularization. Infants fulfilling the inclusion criteria were included over a period of one year (2019–2020). Treatment with topical 0.1% dexamethasone eye drops was started at the first sign of stage 3 ROP. If severe haziness occurred, three drops/day were initiated for three days, tapering to 2 drops for four days, whereafter one drop daily was administered. Patients with stage 3 zone II were given one drop daily until regression to stage 2, median five weeks (range 1–13 weeks), whereafter, one drop every other day was administered for one week. Screening was performed with standardized protocols, classification, and ROP diagnosis were performed according to international screening guidelines[19] once to twice a week with RetCam, for objective analyses, by three experienced ROP screeners (AH, MP, and PL).

Exclusion criteria were infection in the eye, and no patient was excluded.

### Animals

C57BL/6J mice (#000664, Jackson Laboratory) were housed under a 12-hourly light/dark cycle. Pups weighing less than 5.0g or more than 7.5g at P17 were excluded[20]. Both littermate females and males were used. All animal care and experiments were in accordance with the Association for Research in Vision and Ophthalmology Statement for the Use of Animals in Ophthalmic and Vision Research and were approved by the Institutional Animal Care and Use Committee at Boston Children’s Hospital (Protocol Number: 00001619).

### Oxygen-induced retinopathy mouse model and quantification

In mouse OIR, C57BL/6J pups and their nursing dam were placed in a 75% oxygen chamber from P7 to P12 to inhibit retinal vessel growth and induce vessel loss and then returned to room air at P12. Relative hypoxia of avascular retina induces both pathological neovascularization (NV) and re-vascularization (reflected by decreased vaso-obliteration, VO). Mice were euthanized using CO2 asphyxiation or ketamine/xylazine (depending on age) and both eyes were enucleated. After one-hour fixation with 4% paraformaldehyde, retinas were dissected and stained with isolectin GS-IB4 (Alexa Fluor 594, #121413, Invitrogen) in 1 mmol/L CaCl2 in phosphate-buffered saline (PBS, (#10010–023, Gibco) overnight at room temperature to visualize blood vessels. Retinas were washed with PBS and mounted using ProLong Glass Antifade Mountant (#P36980, Invitrogen). Images were taken at 50X magnification using a Zeiss fluorescent microscope. VO and NV were quantified using Image J[21]. The percentages of NV and VO of the total retinal area were calculated and compared between interventions.

#### Treatments.

Dexamethasone sodium phosphate ophthalmic solution (0.1% (1mg/mL)), NDC 24208-720-02, Bausch Lomb Inc.) was topically administered (5 μL) once per day to each eye from P12 to P14 (prior to any neovessel formation), or P14 to P16 (prior to peak neovessel formation) or P17 to P19 (at peak neovessel formation and regression). PBS was topically administered to both eyes of littermates as vehicle control. Oligomycin A (#11342, Cayman Chemical) (0.25μg/g body weight, dissolved in PBS with 20% ethanol) or vehicle control (PBS with 20% ethanol) were intraperitoneally injected in OIR mice from P14 to P16. Retinas were collected at P17 and P20 to examine retinal vascular networks.

### Labe-free LC-MS/MS proteomics

#### Sample preparation:

OIR mice were euthanized at P17 using ketamine/xylazine and retinas were immediately isolated. The two retina from each mouse were pooled for each sample and homogenized in RIPA buffer (#89900, Thermo Fisher Scientific) with protease inhibitor (#P0044, Sigma) and phosphatase inhibitor (#P8340, Sigma). Lysates were proteolyzed using the iST in-solution digestion kit (#P.O.00027, PreOmics GmbH) automated on the PreON robot (PreOmics). In brief, 50 μg retina protein sample in 10 μL was added to 40 μL LYSE buffer (PreOmics). The samples were trypsinized for 3 hours following the manufacturer’s instructions. Eluted peptides were dried in a speed vacuum (Vacufuge, Eppendorf) and resuspended in 40 μL LC-LOAD solution. In total, n = 6 vehicle control and n = 6 dexamethasone samples were prepared.

#### Mass spectrometry:

Mass spectra were acquired on Orbitrap Fusion Lumos coupled to an Easy-nLC1000 HPLC pump (Thermo Fisher Scientific). The peptides were diluted 5-fold using sample loading buffer and 4 ul injections separated using a dual column set-up: an Acclaim^™^ PepMap^™^ 100 C18 HPLC Column, 75 μm X 70 mm (Thermo Fisher Scientific, #164946); and an EASY-Spray^™^ HPLC Column, 75 μm X 250 mm (Thermo Fisher Scientific, #ES902). The column was heated at a constant temperature of 45 °C. The gradient flow rate was 300 nL/min from 5 to 21% solvent B (0.1% formic acid in acetonitrile) for 75 minutes, 21 to 30% solvent B for 15 minutes, and another 10 minutes of a 95%−5% solvent B in a jigsaw wash. Solvent A was 0.1% formic acid in mass spectrometry-grade water. The mass spectrometer was set to 120,000 resolution, and the top N precursor ions in a 3 second cycle time (within a scan range of m/z 400–1500; isolation window, 1.6 m/z) were subjected to collision-induced dissociation (CID, collision energy 30%) for peptide sequencing.

The acquired peptide spectra, comprising 12 retinal samples (n = 6 vehicle control and n = 6 dexamethasone) were searched with the Proteome Discoverer package (PD, Version 2.5) using the SEQUEST-HT search algorithm against the Mouse UniProt database (63,603 entries, updated January 2022). The digestion enzyme was set to trypsin and up to two missed cleavages were allowed. The precursor tolerance was set to 10 ppm and the fragment tolerance window to 0.6 Da. Methionine oxidation and n-terminal acetylation were set as dynamic modifications, and cysteine carbamidomethylation as a static modification. The PD Percolator algorithm calculated the peptide false discovery rate (FDR) and peptides were filtered based on an FDR threshold of 1.0%. Peptides that were only assigned to one given protein group and not detected in any other protein group were considered unique and used for further analyses. A minimum of 2 unique peptides for each protein were required for the protein to be included in the analyses. The Feature Mapper was enabled in PD to quantify peptide precursors detected in the MS1 but may not have been sequenced in all samples. Chromatographic alignment was performed with a maximum retention time shift of 10 minutes, mass tolerance of 10 ppm and signal-to-noise minimum of 5. Precursor peptide abundances were based on their chromatographic intensities and total peptide amount was used for PD normalization.

#### Analysis:

Data were further analyzed using the statistical software, Qlucore (Qlucore, Sweden, version 3.5). We performed a two-group comparison (dexamethasone vs. control eye drops) using the log-transformed protein group means, the student’s t-test for each protein’s comparison (p-value), and the Benjamini-Hochberg procedure to calculate the FDR adjusted p-value (q value). We employed Ingenuity Pathway Analysis (IPA, QIAGEN) to evaluate signaling pathways from gene expression data. IPA calculates Fisher’s exact p-value for overlapping differentially expressed genes with curated gene sets representing canonical biological pathways. In addition, IPA calculates a Z-score for the direction of gene expression for a pathway based on the observed gene expression in the dataset. The Z-score signifies whether expression changes for genes within pathways are consistent with what is expected based on previously published analyses annotated in the Ingenuity Knowledge Base[22]. Significant pathways were defined as those with a Z-score absolute value >1 or an overlap p-value < 0.05. Principal component analysis was performed on unfiltered proteome (p = 1).

### Real-time quantitative PCR (RT-qPCR)

Mice were euthanized at P17 using ketamine/xylazine and retinas were immediately isolated. Total RNA was extracted from pooled retinas of both eyes with PureLinkTM RNA Mini Kit (#12183025, Invitrogen), and cDNA was generated with iScriptTM Reverse Transcription Supermix (#1708841, Bio-Rad). RT-qPCR was performed using SYBR Green qPCR Master Mix (#522076, Bimake.com) and CFX96TM Real-Time PCR Detection System (Bio-Rad, California, USA). Data were quantified using the ΔΔCt method with Cyclophilin A as the internal control **Supplementary Table 1** shows primer sequences of target genes.

### Single-cell RNA sequencing and transcriptome analysis

In the single-cell datasets of “Study - Single-cell RNAseq of Normoxic and OIR mouse retina by Drop-seq” (NCBI’s Gene Expression Omnibus accession no. GSE150703)[23], gene expression of inflammatory markers was analyzed.

### Statistics

All data are presented as the mean ± SEM. The normality and variance of the data set were confirmed using a histogram, normality test, and a quantile-quantile plot. Mann-Whitney U test was used if the data set was not normally distributed. When normality was given, two-tailed unpaired t-test was used when the data set showed equal variance, and Welch’s test was used if the data set had unequal variance (Prism v9.0, GraphPad Software, Inc.). P values < 0.05 were considered statistically significant.

## Results

### A pilot clinical study of topical 0.1% dexamethasone eye drops in Type 2 ROP prior to neovessel formation prevented progression to Type 1 ROP and promoted normal vascular development

**Figure 1:**
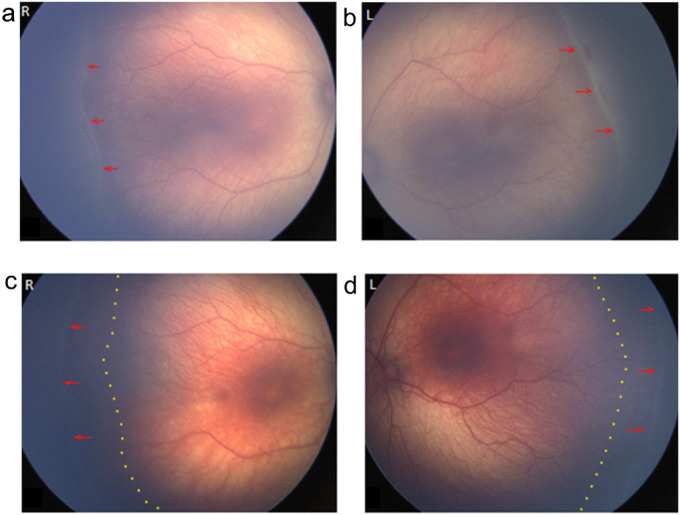
A pilot clinical study of topical 0.1% dexamethasone eye drops Type 2 ROP prior to neovessel formation prevented progression to Type 1 ROP, and promoted normal vascular development. (**a, b**) Representative fundus images of Case 3 (Table 1). Born gestational age 25 weeks, birth weight 700g, female. Start of dexamethasone treatment (a) in right eye (R) and (b) left eye (L) at postmenstrual age (PMA) 36+2 weeks. Red arrows indicate a ridge at the leading edge of developing retinal vasculature. (**c, d**) Representative fundus images of Case 3 (Table 1), at the end of dexamethasone treatment (c) in R eye and (d) L eye at PMA 44+2 weeks. Red arrows indicate the extent of re-vascularization at the end of treatment. Dotted lines indicate the leading edge of vascular development at start of dexamethasone treatment (PMA 36+2).

We conducted a pilot clinical study of five extremely preterm infants (gestational ages at birth from 22 to 27 weeks) with type 2 ROP (stage 3, zone II without plus disease) treated with dexamethasone (0.1%) eye drops during the period 2019–2020. Topical dexamethasone off-label treatment (Fig. 1 a, b) regressed ROP stage 3 lesions and promoted normal vascular development (Fig. 1c, d). In 5/5 clinical cases, topical dexamethasone prevented ROP progression to severe type 1 ROP and prevented the need for laser treatment of retinal neovascularization (Table 1). Potential ocular complications including high intraocular pressure and cataract were not observed in any infants.

### Time-dependent effect of topical 0.1% dexamethasone eye drops on neovascularization in mouse OIR

**Figure 2:**
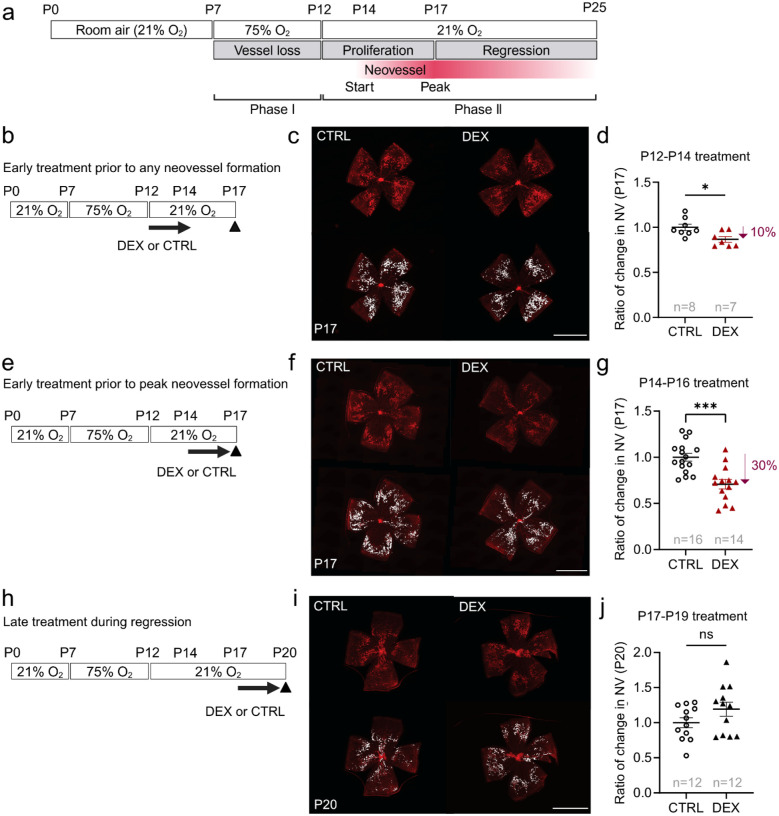
Effect of topical 0.1% dexamethasone eye drops on P17 neovascularization with treatment during different intervals during OIR development. (**a**) Schematic of mouse oxygen-induced retinopathy (OIR) and longitudinal development of neovascularization. Hyperoxia (75% O_2_) from postnatal day (P) 7 to P12 induces retinal vaso-obliteration. After returning to room air (21% O_2_) at P12 the avascular retina drives neovessel formation starting at P14, peaking at P17 with subsequent regression. (**b, e, h**) Schematics of topical 0.1% dexamethasone eye drops (DEX) or control eye drops (CTRL) treatment intervals in OIR. (b) P12 to P14 (prior to any neovessel formation) (e) P14 to P16 (prior to peak neovessel formation) (h) P17 to P19 (during peak neovessel formation and regression). Retinas were collected at P17 (b-g) or P20 (h-j). (**c, f, i**) Representative images of whole mounted retinas after OIR mice were treated with DEX or CTRL were shown. Retinal vessels (red, lectin), neovascular area highlighted in white. Scale bar, 2mm. (**d, g, j**) Ratio of change in neovascularization area (NV) of whole mounted retinas of OIR mice treated topically with DEX vs. CTRL from: (d) P12-P14, evaluated at P17: CTRL, n=8; DEX, n=7 retinas; Two-tailed unpaired t-test; *p<0.05. Mean values ± SEM; (g) P14-P16 evaluated at P17: CTRL, n=15; DEX, n=14 retinas; Two-tailed unpaired t-test; ***p<0.001. (j) P17-P19 evaluated at P20: CTRL, n=12; DEX, n=12 retinas; Two-tailed unpaired t-test or Mann-Whitney U test; ns, not significant.

In mouse OIR, retinal neovessel growth begins at about postnatal day (P) 14, with peak neovessel formation at P17, followed by spontaneous regression between P17 and P25 (Fig. 2a). We applied one drop daily of dexamethasone (0.1%) or control for three days at three key stages in phase II ROP: a) prior to any neovascular formation (P12-P14), b) prior to peak neovessel formation (P14-P16), and c) at peak neovessel formation and subsequent regression (P17-P19).

Topical dexamethasone decreased retinal neovessel formation at P17 by 10% when applied from P12 to P14 (Fig. 2b, c, d) and decreased neovascularization by 30% when applied from P14 to P16 (Fig. 2e, f, g). When applied from P17 to P19, there was a trend to increased P17 neovascularization, which was not statistically significant (Fig. 2h, i, j). Topical dexamethasone did not significantly impact vaso-obliteration (**Supplementary Fig. 1a, b, d, e, g, h**), indicating no suppression of physiological retinal vessel growth. Body weight was comparable in all treatment versus control groups (**Supplementary Fig. 1c, f, i**), suggesting no major toxicity.

### Label-free proteomic analyses of P17 OIR retinas with topical dexamethasone treatment prior to peak neovessel formation

**Figure 3:**
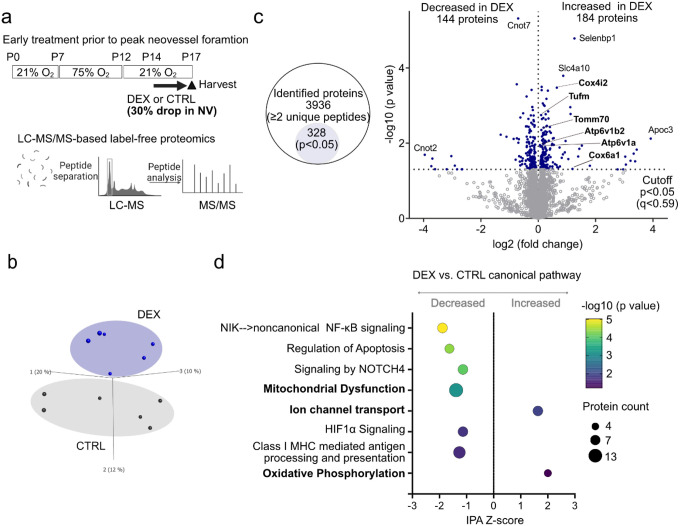
Label-free proteomic analyses of P17 OIR retinas with topical dexamethasone treatment prior to peak neovessel formation (P14-P16). (**a**) Overview of the experimental time course. LC-MS/MS-based label-free proteomics of P17 OIR retinas treated topically with one drop per day of 0.1% DEX or CTRL from P14 to P16 (prior to peak neovessel formation). CTRL, n=6; DEX, n=6 mice (2 retinas from each mouse pooled for n=1). (**b**) Principal component analysis plot of unfiltered proteome (p=1 for n=3,936 proteins; 2 or more unique peptides). (**c**) Number of identified and statistically significant (p<0.05) proteins in the dataset. In DEX group versus CTRL group, 184 proteins with increased and 144 proteins with decreased in abundance. Volcano plot of differentially abundant proteins in P17 OIR DEX vs. CTRL retinas. Each datapoint in blue represents a unique protein considered significant (p<0.05). (**d**) Ingenuity Pathway (IPA) activation Z-scores for selected canonical pathway in DEX group compared to CTRL group. Pathways were sorted by −log10 (p value). Significant pathways were defined as those with a z-score absolute value >1 or an overlap p value <0.05.

To investigate potential pathways modulated by maximum topical dexamethasone suppression of neovascularization in OIR (P14-P16 treatment), we conducted label-free liquid chromatography coupled with mass spectrometry (LC-MS) based proteomics on retinas at P17 (Fig. 3a) as this timing in OIR aligns with clinical results in ROP patients (Fig. 1a–d).

Principal component analysis of the unfiltered proteome (p = 1, q = 1) revealed distinct protein profiles in topical dexamethasone (0.1%) vs. control-treated retinas (Fig. 3b). A total of 3,936 proteins were characterized by at least 2 unique peptides, and 328 proteins had statistically significant changes in abundance (p < 0.05, q < 0.59). Of the significantly altered proteins, 184 had increased intensities and 144 had decreased intensities in the topical dexamethasone- versus control-treated groups (Fig. 3c). We identified differentially activated pathways using Ingenuity Pathway Analysis (IPA)[22] (Fig. 3d). The mitochondria dysfunction pathway was decreased, and Ion channel transport and oxidative phosphorylation pathways were increased in topical dexamethasone-treated OIR retinas. Inflammation, including nuclear factor-kB (NF-kB) signaling and class I MHC mediated antigen processing and presentation, was decreased in dexamethasone-treated OIR retinas, which supports that dexamethasone, a well-known anti-inflammatory agent, influences the retinal proteome.

### Topical dexamethasone treatment (P14-P16) suppressed P17 neovascularization via mitochondrial ATP production in mouse OIR

**Figure 4:**
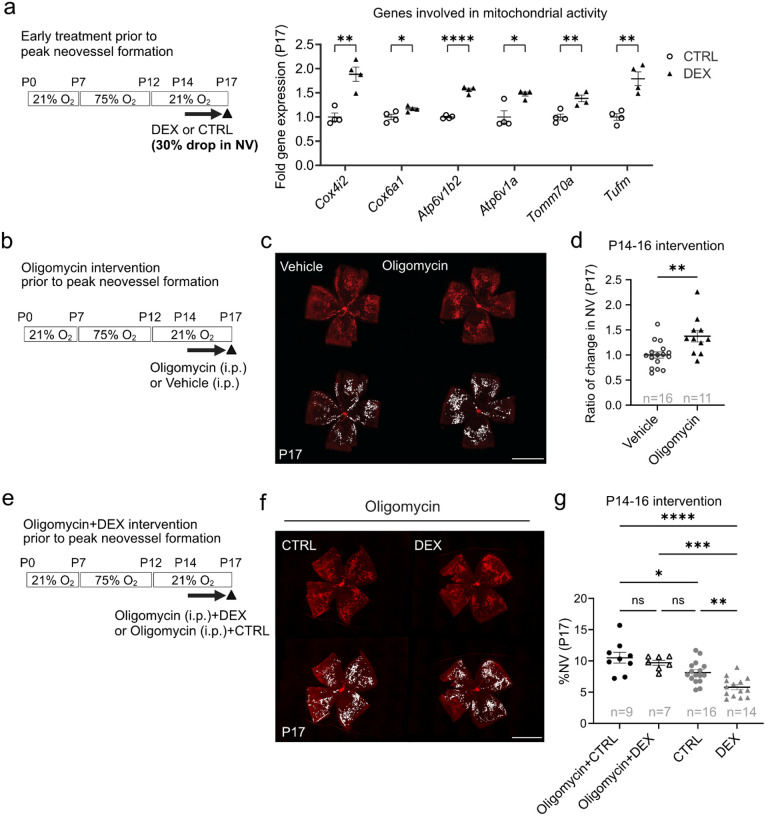
Topical dexamethasone treatment prior to peak neovessel formation (P14-P16) suppresses P17 neovascularization via mitochondrial ATP production. (**a**) Quantification of mitochondrial mRNA gene expression levels in P17 retinas from OIR mice treated with one drop per eye per day of DEX or CTRL from P14 to P16 (prior to peak neovessel formation). CTRL, n=4; DEX, n=4 mice (2 retinas from each mouse pooled for n=1). Two-tailed unpaired t-test or Mann-Whitney U-test; *p<0.05; **p<0.01; ***p<0.001; ****p<0.0001; ns, not significant. Mean values ± SEM. (**b**) Schematic of oligomycin (mitochondrial ATP synthase inhibitor) intervention in OIR from P14 to P16 (prior to peak neovessel formation). (**c**) Representative images of P17 whole mounted retinas from OIR mice after oligomycin (0.25ug/g, i.p., daily) or vehicle P14-P16. Retinal vessels (red, lectin). NV was highlighted in white. Scale bar, 2mm. (**d**) Percent neovascular area of whole retinal area in P17 OIR after oligomycin or control administration in (c). Control, n=16; oligomycin, n=11 retinas; Two-tailed unpaired t-test; **p<0.01; ns, not significant. (**e**) Schematic of intervention in OIR mice treated with oligomycin (0.25ug/g, i.p.) in addition to 0.1% dexamethasone eye drops (oligomycin+DEX) or control eye drops (oligomycin+CTRL) from P14 to P16 (prior to peak neovessel formation). (f) Representative images of whole mounted P17 retinas of OIR mice after treatment with oligomycin+CTRL or oligomycin+DEX from P14 to P16. Retinal vessels are visualized with lectin (red) and NV is highlighted in white. Scale bar, 2mm. (g) Quantification of NV as percentage of total retinal area in P17 OIR retinas in mice treated with oligomycin+CTRL or oligomycin+DEX from P14 to P16. Oligomycin+CTRL, n=9; Oligomycin+DEX, n=7 retinas. DEX and CTRL groups from Figure 2G are included for comparison. One-way ANOVA; *p<0.05; **p<0.01; ***p<0.001; ****p<0.0001; ns, not significant.

We found significantly increased expression of genes associated with mitochondrial activity at P17 in retinas treated with topical dexamethasone eye drops (0.1%) during P14-P16 (Fig. 4a), consistent with the proteomics data (Fig. 3c, d).

We wished to determine if topical dexamethasone eye drops (P14-P16, prior to peak neovessel formation) suppressed P17 neovascularization in OIR mice through increased mitochondrial function. We inhibited mitochondrial ATP FIFO synthetase[24], the final enzyme of oxidative phosphorylation generating ATP from ADP for energy production in the mitochondrial electron transport chain with daily intraperitoneal oligomycin A administration from P14 to P16 (Fig. 4b). Oligomycin alone versus vehicle control significantly increased retinal neovascularization at P17 (Fig. 4c, d) with no significant alteration in retinal vaso-obliteration (**Supplementary Fig. 2a, b**). Oligomycin treatment did not affect body weight (**Supplementary Fig. 2c**), indicating no severe toxicity. To determine if topical dexamethasone eye drops treatment suppressed neovascularization in OIR through increased mitochondrial function, mouse pups were treated with systemic oligomycin in addition to either topical dexamethasone eye drops (DEX + oligomycin) or control eye drops (CTRL + oligomycin) daily from P14 to P16 (Fig. 4e). At P17, neovascularization did not differ between DEX + oligomycin and CTRL + oligomycin groups, suggesting that oligomycin attenuated dexamethasone suppression of neovascularization (Fig. 4f, g). Topical dexamethasone plus oligomycin and control plus oligomycin treatments did not affect vaso-obliteration and body weight (**Supplementary Fig. 2d-f**).

Our results suggested that topical dexamethasone eye drops administered prior to peak neovessel formation (P14-P16) increased mitochondrial function and that blocking mitochondrial ATP synthesis with oligomycin attenuated the suppressive effect of topical dexamethasone eye drops on P17 neovascularization.

### Topical dexamethasone treatment suppressed pro-inflammatory gene expression in OIR retinas in a time-dependent manner

**Figure 5:**
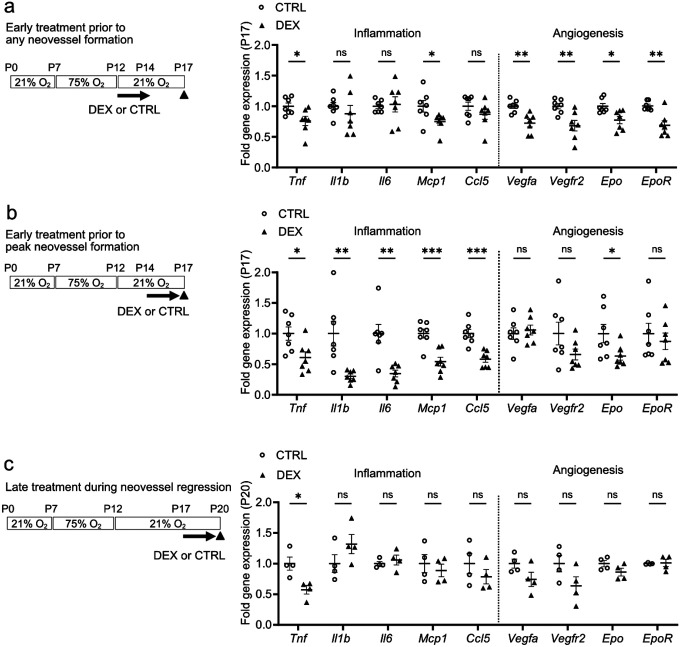
Topical dexamethasone treatment at different at different intervals during OIR progression differentially suppressed pro-inflammatory and pro-angiogenic gene expression in P17 OIR retinas. (**a-c**) Quantification of inflammation- and angiogenesis-related mRNA expression levels in P17 (a, b) or P20 (c) retinas in OIR mice treated with DEX or CTRL (a) from P12 to P14 (prior to any neovessel formation). CTRL, n=7; DEX, n=7 mice (2 retinas from each mouse pooled for n=1); Two-tailed unpaired t-test or Mann-Whitney U-test; *p<0.05; **p<0.01; ns, not significant. Mean values ± SEM. (b) from P14 to P16 (prior to peak neovessel formation). CTRL, n=7–8; DEX, n=8 mice (2 retinas from each mouse pooled for n=1); Two-tailed unpaired t-test or Mann-Whitney U-test; *p<0.05; **p<0.01; ***p<0.001; ns, not significant. Mean values ± SEM. (c) from P17 to P19 (during peak neovessels and neovessel regression). CTRL, n=4; DEX, n=4 mice (2 retinas from each mouse pooled for n=1); Two-tailed unpaired t-test or Mann-Whitney U-test; *p<0.05; ns, not significant. Mean values ± SEM.

We also examined in P17 OIR retinas, the impact of topical dexamethasone eye drops (0.1%) treatment on inflammation and angiogenesis-related gene expression during treatment at three intervals noted above. Dexamethasone treatment from P12 to P14 slightly decreased P17 OIR retinal gene expression levels of pro-inflammatory markers *Tnf* and *Ccl2/Mcp1* (but not *Illb, 116 or Ccl5*), and minimally decreased hypoxia-regulated angiogenesis markers *Vegfa, Vegfr2, Epo*, and *EpoR* (Fig. 5a). Dexamethasone treatment from P14 to P16 decreased more significantly P17 OIR retinal gene expression levels of *Tnf, ll1b, ll6, Ccl2/Mcp1, Ccl5* and modestly suppressed angiogenesis markers *Epo* and did not affect *Vegfa, Vegfr2, or EpoR* (Fig. 5b). Dexamethasone treatment from P17 to P19 decreased P17 OIR retinal gene expression levels of *Tnf* but not *ll1b, ll6, Ccl2/Mcp1, Ccl5* nor angiogenesis markers *Vegfa, Vegfr2, Epo*, and *EpoR* (Fig. 5c). Taken together, topical dexamethasone suppressed inflammation and angiogenesis markers at P17 in OIR depending on the timing of topical dexamethasone treatment. Single-cell analysis of P17 mouse OIR retinas[23] demonstrated that *Tnf, ll1b, Ccl2/Mcp1, and Ccl5* were expressed almost solely in immune cells (**Supplementary Fig. 3**). These findings suggested that topical dexamethasone treatment suppresses neovascularization in OIR retinas by targeting immune cells.

## Discussion

Glucocorticoid effects on ROP have mainly been studied using systemic delivery, and the importance of timing and dose has been suggested. Postnatal systemic glucocorticoids reduce the incidence of severe ROP in some but not all studies[25, 26]. Meta-analysis of 196,264 infants from 63 studies (Pubmed search: “steroid*”, “cortico*”, “betamethasone”, “dexamethasone”) shows that antenatal corticosteroid exposure may decrease ROP severity but not prevent ROP[27]. In a large cohort study of 1,472 neonates with birth weight < 500 g, postnatal steroid exposure is associated with a higher risk of ROP. However, conclusions are limited as the steroid type, delivery route, timing, or dose are not defined[28]. A small retrospective study suggests that dexamethasone eye drops (0.1%) prior to peak neovessel formation (Type 2 ROP) before potential laser therapy markedly inhibits progression to severe neovascularization (Type I ROP) and decreases the need for laser or anti-VEGF treatment[17] aligned with the findings of our small prospective pilot study in extremely preterm infants at very high risk for severe ROP Dexamethasone eye drops delivered prior to the onset of severe neovascularization (Type 2 ROP) prevented progression to severe ROP (Type 1) and promoted normal vascular development in 5/5 infants. In OIR mice modeling ROP, we found that daily 0.1% dexamethasone eye drops administered prior to peak neovessel formation (versus earlier or later intervention) optimally suppressed pathological neovascularization at P17, associated with increased mitochondrial energy production and suppression of inflammation.

Glucocorticoid (dexamethasone) signaling directly regulates mitochondrial transcription via local mitochondrial glucocorticoid response elements (GREs) and glucocorticoid receptors[29, 30]. Short-term glucocorticoid exposure may induce mitochondrial biogenesis and increase respiratory chain activity. In contrast, long-term glucocorticoid exposure may cause respiratory chain dysfunction, decrease energy production, and increase reactive oxidative stress and mitochondrial structural abnormalities[30–32]. Adverse effects of prolonged and high-dose dexamethasone include impaired glucose clearance and disturbed fatty acid metabolism[33–38]. Therefore, the dose and timing of dexamethasone delivery in premature infants need to be tightly controlled. Topical delivery is a safer approach for treating eye diseases, and our findings suggest that topical dexamethasone has a time-dependent inhibitory effect on neovascularization. The maximum impact of dexamethasone on neovascularization correlated with dexamethasone-induced increased mitochondrial activity. Inhibition of mitochondrial energy production by oligomycin A attenuated dexamethasone suppression of neovascularization. We propose that topical dexamethasone improves mitochondrial function and thereby prevents severe neovascular ROP. We also observed decreased gene expression of pro-inflammatory markers with optimally timed dexamethasone suppression of neovascularization. Inflammation is a significant contributor to neovessel growth in OIR[39, 40]. Interestingly, dexamethasone did not impact inflammation if administered during neovascular regression, confirming the importance of timing topical dexamethasone treatment.

Our observations suggest a link between improved mitochondrial function and suppression of inflammation, possibly through immune cells. Among immune cells, microglia/macrophages are the potential source of retinal cytokines/chemokines and proangiogenic factors[41]. Anti-inflammatory myeloid cells use oxidative phosphorylation (with limited glycolysis). Activation of microglia/macrophages to an inflammatory phenotype is accompanied by a metabolic switch from oxidative phosphorylation to aerobic glycolysis[42]. There is also a profound increase in fatty acid oxidation and mitochondrial biogenesis gene expression in anti-inflammatory macrophages[43]. A switch to aerobic glycolysis accompanied by microglia activation occurs in several neurodegenerative diseases (stroke, Parkinson’s and Alzheimer’s diseases)[44–46]. Blocking glycolysis in activated microglia reduces pro-inflammatory responses[47, 45] and triggers metabolic shifts to fatty acid oxidation, increased ATP production, and high phagocytic activity in brain microglia[48]. A recent single-cell transcriptomics analysis in OIR retinas has identified a cluster of activated microglia that are more glycolytic[49]. Loss of critical glycolytic enzymes *Pfkfb3 or Pkm2 in* myeloid cells decreases neovascularization in mouse OIR[50, 49]. Compromised mitochondrial function leads to increased systemic inflammation and macrophage activation in mice with global deficiency of mitochondrial complex I[51]. We speculate that increased mitochondrial function in myeloid cells after treatment with dexamethasone causes a metabolic switch to a quiescent non-inflammatory phenotype. Further studies are needed to validate this hypothesis.

Our current work has limitations. Strengthening our findings in the current small pilot investigation requires a larger, randomized prospective clinical study. In addition, in the clinical study, topical dexamethasone caused regression of neovessels and promoted normal vessel development, whereas in the OIR mouse model, neovascularization was suppressed, but revascularization did not incrase. This difference may be attributed to variations in treatment duration (weeks in clinical settings versus three days in OIR), the relative size of the eyes in mice compared to humans, or other mechanisms not elucidated in this study. Moreover, further investigations are needed to clarify the underlying mechanisms behind dexamethasone modulation of mitochondrial function and inflammation in myeloid cells.

In summary, we found that topical 0.1% dexamethasone eye drops during early neovessel formation suppressed pathological neovascularization in phase II ROP through modulation of mitochondrial activity. This study provides new therapeutic strategies for ROP and new insights into mitochondrial function in Phase II ROP.

## Figures and Tables

**Table 1:** Dexamethasone eye drops in five extremely preterm infants with Type 2 ROP prevented Type 1 ROP

Pt.	GA (w + d)	BW (g)	Sex	Indication for treatment	DEX (0.1%) dose	DEX start PMA (w + d)	DEX end PMA (w+d)	Post-treatment results
**1**	22 + 4	430	M	ROP Type 2 (stage 3, zone II, no plus), haze	1drop 3x/d x 4d, 1drop 2x/d x 3d, 1drop/d x 4d	33 + 0	34 + 4	Regression of ROP, vascularization to zone 3, less haze
**2**	24 + 3	720	M	ROP Type 2 (stage 3, zone II, no plus)	1drop/d x 1d, 1 drop qod	34 + 3	47 + 6	Regression of ROP, vascularization to zone 3, less haze
**3**	25 + 0	700	F	ROP Type 2 (stage 3, zone II, no plus)	1drop/d x 1d, 1 drop qod	36 + 2	44 + 2	Regression of ROP, vascularization to zone 3, less haze
**4**	27 + 4	565	F	ROP Type 2 (stage 3, zone II, no plus)	1drop/d x 1d, 1 drop qod	36 + 4	41 + 4	Regression of ROP, vascularization to zone 3, less haze
**5**	25 + 4	750	M	ROP Type 2 (stage 3, zone II, no plus)	1drop/d x 1d, 1 drop qod	37 + 1	39 + 4	Regression of ROP, vascularization to zone 3, less haze

Abbreviations: Pt., patient, GA, gestational age; w, weeks; d, days; BW, birth weight; M, male; F, female; DEX, dexamethasone eyedrops; PMA, postmenstrual age; qod, every other day.

## Data Availability

All the data supporting the conclusions of this study are included within the article and supplementary data. All the other data and materials are available upon request to the corresponding author. The mass spectrometry proteomics data have been deposited to the ProteomeXchange Consortium via the PRIDE[52] partner repository with the dataset identifier PXD052730 and 10.6019/PXD052730.

## Abbreviations

ATP: Adenosine triphosphate
CCL5: CC motif chemokine lignad 5
CTRL: Control
DEX: Dexamethasone
EPO: Erythropoietin
EPOR: Erythropoietin receptor
ll-1β: Interleukin-1 beta
Il-6: Interleukin-6
LC-MS: label-free liquid chromatography coupled with mass spectrometry
MCP1: Monocyte chemoattractant protein-1
NF-kB: Nuclear factor kappa B
NV: Neovascularization
OIR: Oxygen-induced retinopathy
P: Postnatal day
ROP: Retinopathy of prematurity
TNF: Tumor necrosis factor
VEGFA: Vascular endothelial growth factor A
VEGFR: Vascular endothelial growth factor receptor
VO: Vaso-obliteration
